# Synthesis, crystal structure and Hirshfeld surface analysis of a polymeric bis­muthate(III) halide complex, (C_6_H_6_N_3_)_2_[BiCl_5_]·2H_2_O

**DOI:** 10.1107/S2056989017015134

**Published:** 2017-10-24

**Authors:** Chaima Boukoum, Zouhaier Aloui, Valeria Ferretti, Sonia Abid

**Affiliations:** a Laboratoire de Chimie des Matériaux, Faculté des Sciences de Bizerte, 7021 Zarzouna Bizerte, Tunisia; b Department of Chemical and Pharmaceutical Sciences, Centre for Structural, Diffractometry, University of Ferrara, Via L. Borsari 46, I-44121 Ferrara, Italy

**Keywords:** crystal structure, halogenobismuthates, benzotriazole, Hirshfeld surface analysis

## Abstract

The synthesis and the crystal structure of a new halide-bridged polymer, namely *catena*-poly[bis­(1,2,3-benzotriazolium) [[tetra­chlorido­bis­muth(III)]-μ-chlorido] dihydrate], {(C_6_H_6_N_3_)_2_[BiCl_5_]·2H_2_O}_*n*_ are reported. The structure comprises polyanionic zigzag chains of formula [(BiCl_5_)^2−^]_*n*_ running along the *c-*axis direction. The 1,2,3-benzotriazolium cations are linked between these polymer chains, *via* the water mol­ecules, giving rise to left- and right-handed helical chains. Hirshfeld surface analysis and fingerprint plots were used to decode the inter­molecular inter­actions in the crystal network and determine the contribution of the component units for the construction of the three-dimensional architecture.

## Abstract 2

The structure of this new halide-bridged polymer comprises polyanionic zigzag chains of formula [(BiCl_5_)^2−^]_*n*_ running along the *c-*axis direction. The 1,2,3-benzotriazolium cations are linked between these polymer chains, *via* the water mol­ecules, giving rise to left- and right-handed helical chains.

## Chemical context   

Bismuth–halide complexes are of contemporary inter­est because of their structural diversity and numerous promising physical properties such as dielectric, ferroelectric, ferro­elastic, non-linear optical and thermochromism (Bator *et al.*, 1997 ▸; Bednarska-Bolek *et al.*, 2000 ▸; Sobczyk *et al.*, 1997 ▸; Bator *et al.*, 1998 ▸). Generally, in these compounds, the Bi*X*_6_ octa­hedra may join to form discrete (*i.e*. mononuclear) or extended (*i.e*. polynuclear) inorganic networks of corner-, edge-, or face-sharing octa­hedra, leading to an extensive family of bis­muth halogenoanions (Jakubas, 1986 ▸; Jakubas *et al.*, 1988 ▸, 1995 ▸). A variety of organic cations, ring shaped or linear, have a strong impact on the arrangements of Bi*X*_6_ octa­hedra and the formation of hydrogen bonds (Dammak *et al.*, 2015 ▸; Elfaleh & Kamoun, 2014 ▸). This class of compounds has also attracted much attention in the field of crystal engin­eering over the last decade on account of their capability for the creation of extended architectures *via* inter­molecular non-covalent binding inter­actions. (*i.e.* hydrogen bonding, ionic and π–π stacking inter­actions; Belter & Fronczek, 2013 ▸; Thirunavukkarasu *et al.*, 2013 ▸; Aloui *et al.*, 2015 ▸).
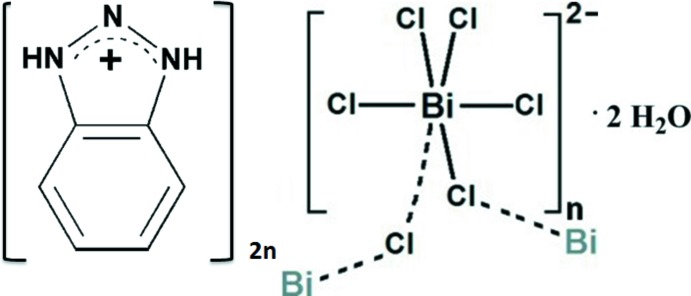


As part of our studies in this area, we chose benzotriazole, which is an aromatic heterocyclic base with three protonatable nitro­gen atoms, as the organic cation.

## Structural commentary   

**Figure 1:**
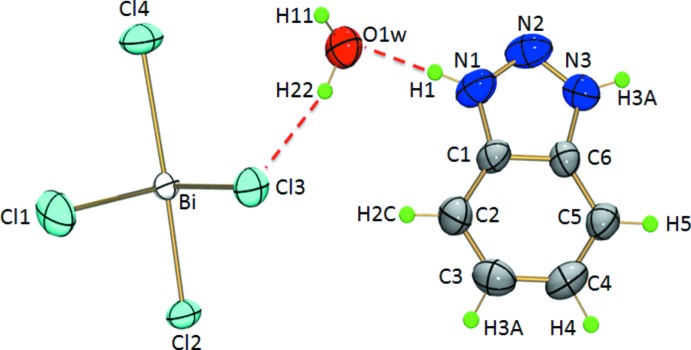
The asymmetric unit of (I) showing 50% displacement ellipsoids.

The single-crystal X-ray diffraction analysis shows that the title compound [C_6_H_6_N_3_]_2_[BiCl_5_]·2H_2_O, (I), crystallizes in the non-centrosymmetric space group *Cmc*2_1_ and the asymmetric unit comprises one Bi^3+^ cation, four chlorine atoms, one water mol­ecule and one benzotriazolium cation (Fig. 1 ▸). The bis­muth atom is six-coordinated by four distinct chlorine atoms (Cl1, Cl2, Cl3, Cl4). The Bi—Cl bond lengths (Table 1 ▸) vary from 2.545 (3) to 2.674 (4) Å (ΔBi—Cl = 0.129 Å) and 2.757 (4) to 2.856 (4) Å (ΔBi—Cl = 0.099 Å) for non-bridging and bridging Cl atoms, respectively, which are comparable with values found in {(C_2_H_7_N_4_O)_2_[BiCl_5_]_*n*_ (Ferjani *et al.*, 2012 ▸) and [NH_3_(CH_2_)_6_NH_3_]BiCl_5_ (Ouasri *et al.*, 2013 ▸). The Cl—Bi—Cl bond angles in (I) range from 85.93 (17) to 91.88 (13)° (ΔCl—Bi—Cl =5.95°) and are less distorted than those observed in [NH_3_(CH_2_)_6_NH_3_]BiCl_5_ and [H_2_mdap][BiCl_5_] (Ouasri *et al.*, 2013 ▸; Wang *et al.*, 2017 ▸).

**Figure 2:**
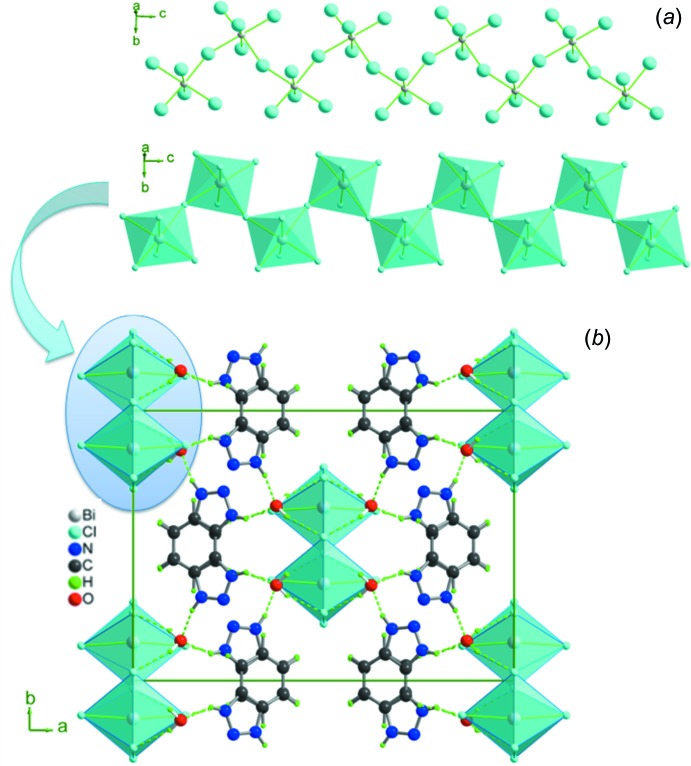
(*a*) View of the [(BiCl_5_)^2−^]_*n*_ polyanionic zigzag chains in (I) along the *c-*axis direction. (*b*) Projection along the *c* axis of the structure of (I).

In the extended structure of (I), adjacent BiCl_6_ octa­hedra are connected through Cl4 and Cl4^iii^ so as to form [(BiCl_5_)^2−^]_*n*_ polyanionic zigzag chains propagating along the c-axis direction, with the shortest intra­chain Bi⋯Bi distance of 5.508 (1) Å and a Cl4—Bi—Cl4^ii^ angle of 89.61 (3)° (Fig. 2 ▸) The overall negative charges of the resulting polymers are counter-balanced by the protonated 1,2,3-benzotriazolium cations (Fig. 2 ▸ *b*). As usual, this aromatic amine is protonated at the N3 atom and the C—C, N—N and C—N bond lengths vary from 1.358 (18) to 1.402 (15), 1.293 (15) to 1.308 (15) Å and 1.364 (16) to 1.370 (15) Å, respectively, which agree well with those observed in bis­(1,2,3-benzotriazolium) sulfate dihydrate (Randolph *et al.*, 2013 ▸) and benzotriazolium picrate (Zeng *et al.*, 2011 ▸).

## Supra­molecular features   

**Figure 3:**
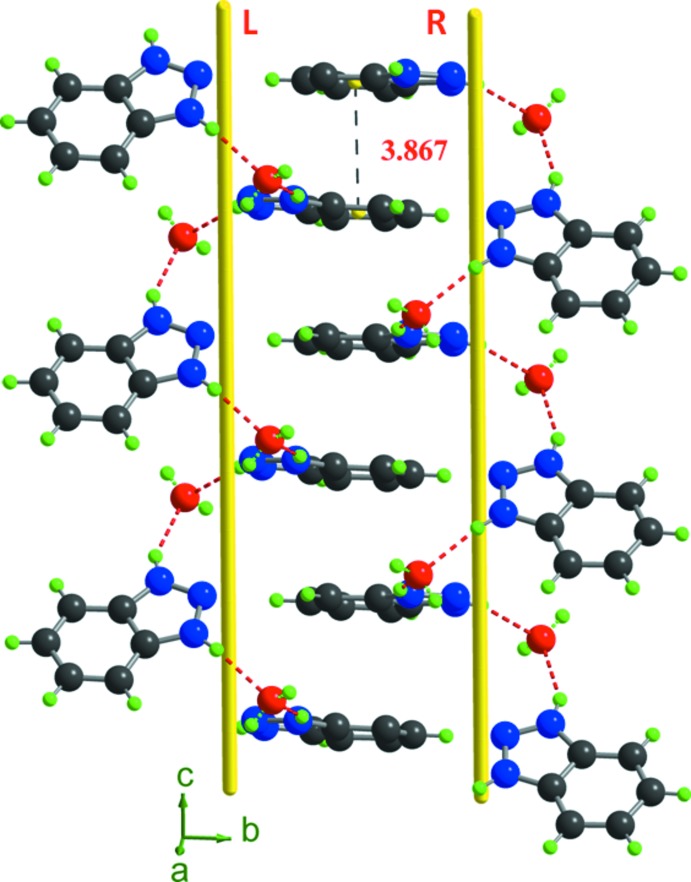
View of the infinite helical hydrogen-bonded chain in (I).

The heterocyclic cations alternately bridge the water mol­ecules (O1*W*) *via* N—H⋯O hydrogen bonds, forming (benzo-O*W*)_*n*_ helical chains in a right- and left–handed sequence extending along the *c*-axis direction (Table 2 ▸, Fig. 2 ▸). The phenyl rings of adjacent chains are alternately stacked in a parallel-displaced face-to-face arrangement (Fig. 3 ▸), with centroid–centroid distances of 3.8675 (1) Å and an inter-planar spacing of 1.13 Å. The anionic and cationic chains are further assembled into a three-dimensional supra­molecular framework through N—H⋯O, O—H⋯Cl and C—H⋯Cl hydrogen bonds (Table 2 ▸, Fig. 3 ▸).

## Hirshfeld surface analysis   

**Figure 4:**
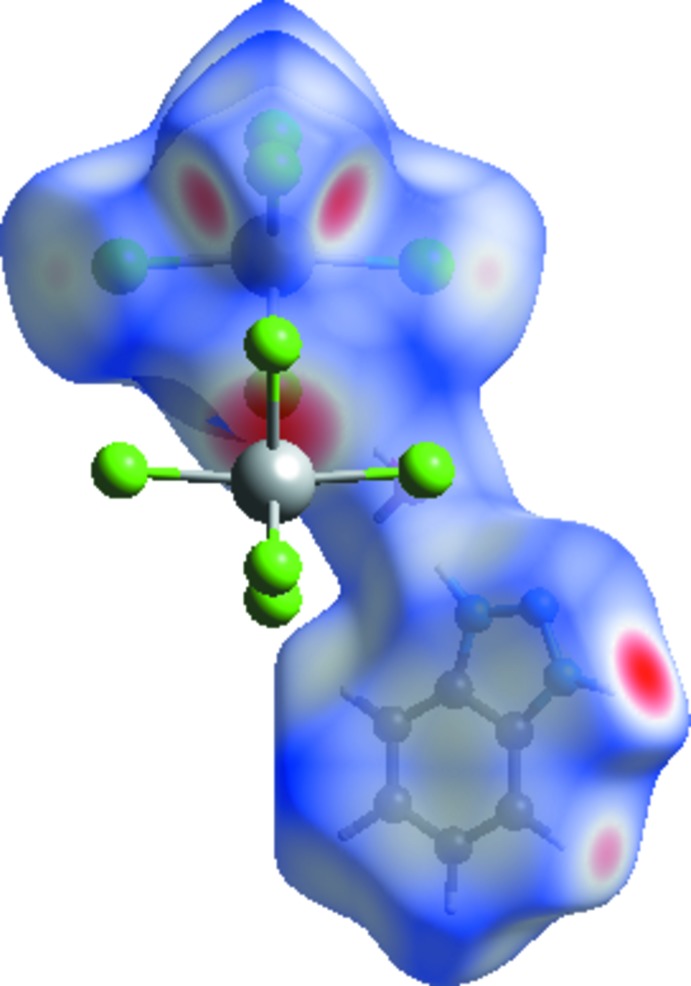
Hirshfeld surface mapped over *d*_norm_ of (I).

**Figure 5:**
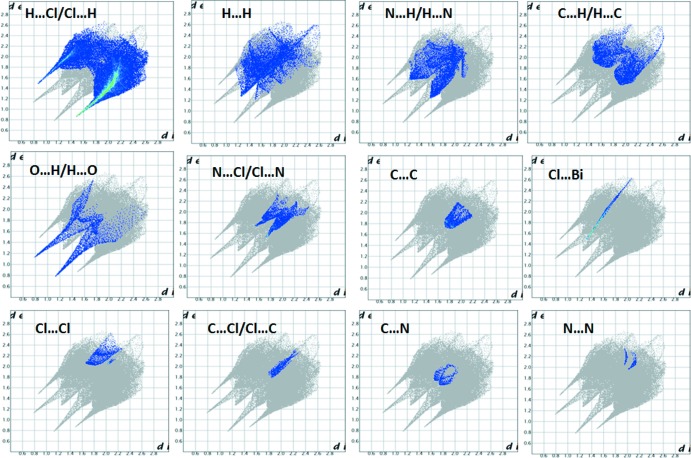
Two-dimensional fingerprint plots for (I) showing contributions from different contacts.

**Figure 6:**
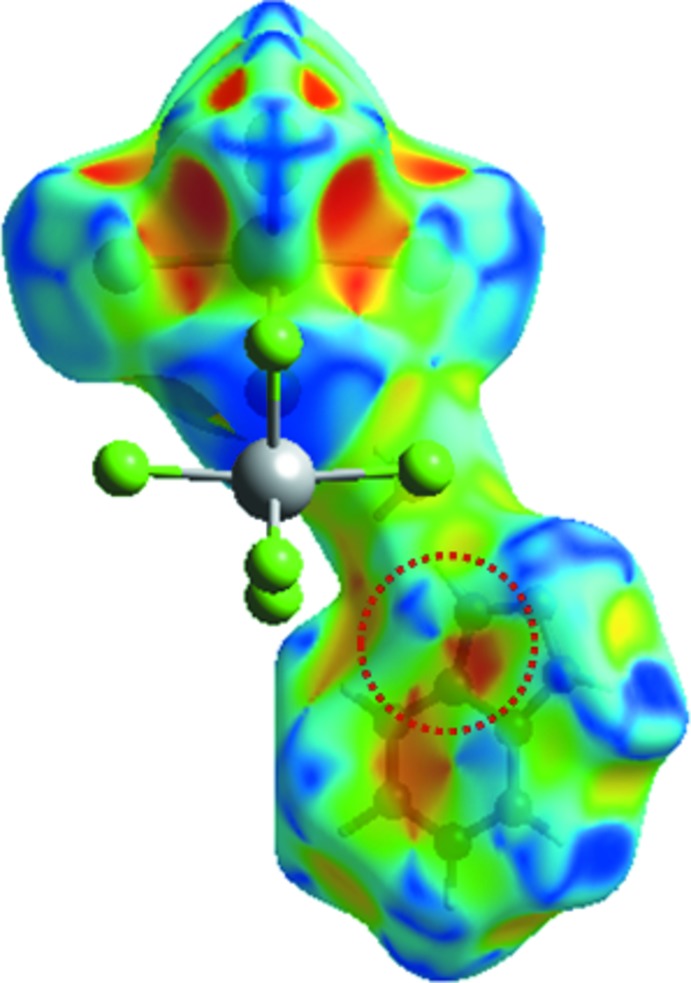
Hirshfeld surface mapped over the shape index for (I) highlighting the regions involved in π-π stacking inter­actions.

The Hirshfeld surface (Wolff *et al.*, 2012 ▸) mapped with a *d*_norm_ function for the asymmetric unit for the title compounds clearly shows the red spots derived from H⋯O and H⋯Cl/Cl⋯H contacts (Fig. 4 ▸). The two-dimensional fingerprint plot shows that the H⋯Cl/Cl⋯H contacts associated with O—H⋯Cl hydrogen bonding appear to be the major contributor in the crystal packing (55.8%): these contacts are represented as regions in the top left (*d*_e_ > *d*_i_, Cl⋯H) and bottom right (*d*_e_ < *d*_i_, H⋯Cl) of the related plots in Fig. 5 ▸. Inter­actions of the type H⋯H appear in the middle of the scattered points in the fingerprint maps; they comprise 10.9% of the entire surface. The decomposition of the fingerprint plot shows that N⋯H/H⋯N, C⋯H/H⋯C, O⋯H/H⋯O and N⋯Cl/Cl⋯N contacts have percentage contributions of 7.8%, 6.5%, 4.5% and 4.3% respectively, of the total Hirshfeld surface. The C⋯C contacts associated with π–π inter­actions amount to 3.4% of the surface: their presence is indicated by the appearance of red and blue triangles on the shape-indexed surfaces in Fig. 6 ▸. The Cl⋯Bi/Bi⋯Cl (3%) inter­actions are represented as points in the top area. The Cl⋯Cl, C⋯Cl/Cl⋯C, C⋯N, and N⋯N inter­actions are in the middle of the fingerprint plots, and comprise a very small contribution of 1.3%, 1.2%, 0.9% and 0.4%, respectively.

The inter­molecular inter­actions were further evaluated by using the enrichment ratio (ER; Jelsch *et al.*, 2014 ▸). The largest contribution to the Hirshfeld surface is from H⋯Cl/Cl⋯H contacts associated with O—H⋯Cl hydrogen bonds and their ER value is 1.73. The H⋯H contacts are the second largest contributor, but they display an enrichment ratio significantly below unity (ER_HH_ = 0.47). The formation of extensive π–π inter­actions is reflected in the relatively high ER_CC_ of 3.94.

## Synthesis and crystallization   

The title compound was prepared by dropwise addition of an ethano­lic solution of 1*H*-benzotriazole (0.061 g, 0.5 mmol) to 1 mmol of a bis­muth nitrate solution [Bi(NO_3_)_3_·5H_2_O], dissolved in 0.05 mL of a concentrated HCl aqueous solution. The resulting aqueous solution was stirred for 30 min. and kept at room temperature for crystallization. After two week of slow evaporation, colourless single crystals of (I) (yield = 75%) were formed in the solution. Analysis observed (calculated) for [C_6_H_6_N_3_]_2_[BiCl_5_]·2H_2_O (%): C 21.6 (21.0), H 2.66 (2.41), N 34.6 (33.8).

## Refinement   

Crystal data, data collection and structure refinement details are summarized in Table 3 ▸. The N-bound and C-bound hydrogen atoms were positioned geometrically and treated as riding: N—H = 0.86 Å and C—H = 0.93 Å with *U*_iso_(H) = 1.2*U*_eq_(N,C). The O—H and H⋯H separations in the water mol­ecule were restrained using a DFIX model to be 0.90 and 1.46 Å, respectively, and refined with *U_iso_*(H) = 1.5*U*_eq_(O).

## Supplementary Material

Crystal structure: contains datablock(s) I, New_Global_Publ_Block. DOI: 10.1107/S2056989017015134/hb7714sup1.cif

Structure factors: contains datablock(s) I. DOI: 10.1107/S2056989017015134/hb7714Isup2.hkl

CCDC reference: 1580458

Additional supporting information:  crystallographic information; 3D view; checkCIF report

## Figures and Tables

**Table 1:** Selected bond lengths (Å)

Bi1—Cl1	2.669 (3)	Bi1—Cl4^i^	2.757 (4)
Bi1—Cl2	2.545 (3)	Bi1—Cl4	2.856 (4)
Bi1—Cl3	2.674 (4)		

Symmetry code: (i) 
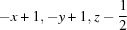
.

**Table 2:** Hydrogen-bond geometry (Å, °)

*D*—H⋯*A*	*D*—H	H⋯*A*	*D*⋯*A*	*D*—H⋯*A*
N1—H1⋯O1*W*	0.86	2.08	2.891 (17)	157
N3—H3*A*⋯O1*W* ^ii^	0.86	2.00 (2)	2.767 (18)	148
O1*W*—H11⋯Cl4^iii^	0.89 (11)	2.47 (11)	3.306 (13)	157 (9)
O1*W*—H22⋯Cl3	0.90 (12)	2.38 (12)	3.268 (14)	169 (11)
C5—H5⋯Cl1^iv^	0.93	2.73	3.603 (14)	157

Symmetry codes: (ii) 
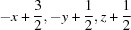
; (iii) 
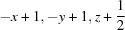
; (iv) 
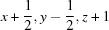
.

**Table 3:** Experimental details

Crystal data	
Chemical formula	(C_6_H_6_N_3_)_2_[BiCl_5_]·2H_2_O
*M*_r_	662.54
Crystal system, space group	Orthorhombic, *C* *m* *c*2_1_
Temperature (K)	293
*a*, *b*, *c* (Å)	19.4627 (4), 13.8181 (4), 7.7343 (2)
*V* (Å^3^)	2080.04 (9)
*Z*	4
Radiation type	Mo *K*α
μ (mm^−1^)	9.14
Crystal size (mm)	0.55 × 0.34 × 0.23

Data collection	
Diffractometer	Nonius KappaCCD
Absorption correction	Multi-scan (*SORTAV*; Blessing, 1995 ▸)
*T*_min_, *T*_max_	0.011, 0.053
No. of measured, independent and observed [*I* > 2σ(*I*)] reflections	6050, 1670, 1643
*R*_int_	0.067
(sin θ/λ)_max_ (Å^−1^)	0.581

Refinement	
*R*[*F* ^2^ > 2σ(*F* ^2^)], *wR*(*F* ^2^), *S*	0.038, 0.096, 1.11
No. of reflections	1670
No. of parameters	131
No. of restraints	4
H-atom treatment	H atoms treated by a mixture of independent and constrained refinement
Δρ_max_, Δρ_min_ (e Å^−3^)	1.68, −0.76
Absolute structure	Flack (1983 ▸), 731 Friedel pairs
Absolute structure parameter	−0.036 (14)

Computer programs: Kappa CCD server software (Nonius, 1997 ▸), *DENZO-SMN* (Otwinowski & Minor, 1997 ▸), *SHELXS97*, *SHELXL97* and *SHELXTL* (Sheldrick, 2008 ▸), *ORTEPIII* (Burnett & Johnson, 1996 ▸), and *WinGX* (Farrugia, 2012 ▸).

## supplementary crystallographic information

### Crystal data

**Table:**

(C_6_H_6_N_3_)_2_[BiCl_5_]·2H_2_O	*F*(000) = 1256
*M**_r_* = 662.54	*D*_x_ = 2.116 Mg m^−^^3^
Orthorhombic, *C**m**c*2_1_	Mo *K*α radiation, λ = 0.71073 Å
Hall symbol: C 2c -2	Cell parameters from 8027 reflections
*a* = 19.4627 (4) Å	θ = 4.4–7.3°
*b* = 13.8181 (4) Å	µ = 9.14 mm^−^^1^
*c* = 7.7343 (2) Å	*T* = 293 K
*V* = 2080.04 (9) Å^3^	Rod, colourless
*Z* = 4	0.55 × 0.34 × 0.23 mm

### Data collection

**Table:**

Nonius KappaCCD diffractometer	1670 independent reflections
Radiation source: fine-focus sealed tube	1643 reflections with *I* > 2σ(*I*)
Graphite monochromator	*R*_int_ = 0.067
f scans and w scans	θ_max_ = 24.4°, θ_min_ = 4.4°
Absorption correction: multi-scan (SORTAV; Blessing, 1995)	*h* = −22→22
*T*_min_ = 0.011, *T*_max_ = 0.053	*k* = −16→16
6050 measured reflections	*l* = −8→8

### Refinement

**Table:**

Refinement on *F*^2^	Hydrogen site location: inferred from neighbouring sites
Least-squares matrix: full	H atoms treated by a mixture of independent and constrained refinement
*R*[*F*^2^ > 2σ(*F*^2^)] = 0.038	*w* = 1/[σ^2^(*F*_o_^2^) + (0.0704*P*)^2^ + 2.8002*P*] where *P* = (*F*_o_^2^ + 2*F*_c_^2^)/3
*wR*(*F*^2^) = 0.096	(Δ/σ)_max_ = 0.038
*S* = 1.11	Δρ_max_ = 1.68 e Å^−^^3^
1670 reflections	Δρ_min_ = −0.76 e Å^−^^3^
131 parameters	Extinction correction: SHELXL97 (Sheldrick, 2008), Fc^*^=kFc[1+0.001xFc^2^λ^3^/sin(2θ)]^-1/4^
4 restraints	Extinction coefficient: 0.0028 (4)
Primary atom site location: structure-invariant direct methods	Absolute structure: Flack (1983), 731 Friedel pairs
Secondary atom site location: difference Fourier map	Absolute structure parameter: −0.036 (14)

### Special details

**Table:**

Geometry. All esds (except the esd in the dihedral angle between two l.s. planes) are estimated using the full covariance matrix. The cell esds are taken into account individually in the estimation of esds in distances, angles and torsion angles; correlations between esds in cell parameters are only used when they are defined by crystal symmetry. An approximate (isotropic) treatment of cell esds is used for estimating esds involving l.s. planes.
Refinement. Refinement of F^2^ against ALL reflections. The weighted R-factor wR and goodness of fit S are based on F^2^, conventional R-factors R are based on F, with F set to zero for negative F^2^. The threshold expression of F^2^ > 2sigma(F^2^) is used only for calculating R-factors(gt) etc. and is not relevant to the choice of reflections for refinement. R-factors based on F^2^ are statistically about twice as large as those based on F, and R- factors based on ALL data will be even larger.

### Fractional atomic coordinates and isotropic or equivalent isotropic displacement parameters (Å^2^)

**Table:**

	*x*	*y*	*z*	*U*_iso_*/*U*_eq_	
Bi1	0.5000	0.35812 (2)	0.08277 (6)	0.0310 (2)	
Cl1	0.36329 (14)	0.3716 (2)	0.0695 (12)	0.0685 (10)	
Cl2	0.5000	0.2105 (2)	−0.1139 (6)	0.0458 (7)	
Cl3	0.5000	0.2501 (3)	0.3697 (5)	0.0500 (8)	
Cl4	0.5000	0.5302 (3)	0.2874 (5)	0.0652 (10)	
O1W	0.6246 (4)	0.3531 (5)	0.585 (3)	0.0570 (18)	
C1	0.6621 (5)	0.0963 (7)	0.7492 (14)	0.042 (2)	
C2	0.6058 (5)	0.0522 (8)	0.6684 (15)	0.049 (2)	
H2C	0.5704	0.0873	0.6175	0.058*	
C3	0.6071 (6)	−0.0470 (8)	0.6707 (16)	0.054 (3)	
H3	0.5705	−0.0804	0.6215	0.065*	
C4	0.6607 (6)	−0.1001 (8)	0.7433 (16)	0.056 (2)	
H4	0.6590	−0.1673	0.7390	0.067*	
C5	0.7154 (7)	−0.0568 (8)	0.8201 (15)	0.047 (3)	
H5	0.7511	−0.0926	0.8684	0.057*	
C6	0.7153 (6)	0.0439 (9)	0.8229 (14)	0.040 (2)	
N3	0.7585 (5)	0.1125 (8)	0.8909 (15)	0.050 (2)	
H3A	0.7954	0.0992	0.9469	0.060*	
N1	0.6790 (5)	0.1908 (7)	0.7770 (15)	0.058 (2)	
H1	0.6545	0.2390	0.7434	0.070*	
N2	0.7371 (5)	0.1995 (8)	0.8609 (16)	0.061 (3)	
H11	0.600 (5)	0.400 (8)	0.638 (18)	0.091*	
H22	0.595 (5)	0.320 (10)	0.520 (18)	0.091*	

### Atomic displacement parameters (Å^2^)

**Table:**

	*U*^11^	*U*^22^	*U*^33^	*U*^12^	*U*^13^	*U*^23^
Bi1	0.0321 (3)	0.0286 (3)	0.0323 (3)	0.000	0.000	−0.0007 (2)
Cl1	0.0362 (11)	0.0924 (19)	0.077 (3)	0.0003 (11)	0.008 (2)	−0.013 (2)
Cl2	0.0575 (19)	0.0308 (16)	0.0490 (17)	0.000	0.000	−0.0089 (14)
Cl3	0.0605 (19)	0.0469 (19)	0.0427 (16)	0.000	0.000	0.0107 (16)
Cl4	0.088 (3)	0.053 (2)	0.054 (2)	0.000	0.000	−0.0215 (16)
O1W	0.042 (3)	0.060 (5)	0.069 (5)	0.002 (2)	0.002 (10)	−0.010 (5)
C1	0.041 (5)	0.033 (5)	0.051 (5)	0.001 (4)	0.009 (4)	−0.002 (4)
C2	0.047 (5)	0.050 (6)	0.049 (5)	0.008 (4)	0.001 (4)	−0.002 (4)
C3	0.048 (5)	0.056 (7)	0.059 (6)	−0.008 (5)	−0.001 (5)	−0.011 (5)
C4	0.064 (6)	0.042 (6)	0.062 (6)	−0.004 (5)	0.016 (5)	−0.005 (5)
C5	0.053 (7)	0.042 (6)	0.047 (6)	0.011 (5)	0.002 (5)	0.001 (5)
C6	0.032 (5)	0.046 (5)	0.040 (5)	0.004 (4)	0.002 (4)	−0.007 (5)
N3	0.044 (5)	0.053 (6)	0.053 (6)	−0.002 (5)	0.005 (4)	−0.006 (4)
N1	0.052 (5)	0.037 (5)	0.085 (7)	−0.001 (4)	0.011 (5)	−0.005 (5)
N2	0.053 (6)	0.050 (6)	0.080 (7)	−0.016 (5)	0.010 (5)	−0.018 (5)

### Geometric parameters (Å, º)

**Table:**

Bi1—Cl1	2.669 (3)	C2—H2C	0.9300
Bi1—Cl1^i^	2.669 (3)	C3—C4	1.394 (17)
Bi1—Cl2	2.545 (3)	C3—H3	0.9300
Bi1—Cl3	2.674 (4)	C4—C5	1.358 (18)
Bi1—Cl4^ii^	2.757 (4)	C4—H4	0.9300
Bi1—Cl4	2.856 (4)	C5—C6	1.392 (13)
Cl4—Bi1^iii^	2.757 (4)	C5—H5	0.9300
O1W—H11	0.90 (2)	C6—N3	1.370 (15)
O1W—H22	0.90 (2)	N3—N2	1.293 (15)
C1—N1	1.364 (16)	N3—H3A	0.8600
C1—C6	1.387 (16)	N1—N2	1.308 (15)
C1—C2	1.402 (15)	N1—H1	0.8600
C2—C3	1.371 (16)		
			
Cl2—Bi1—Cl1	91.88 (13)	C3—C2—H2C	122.8
Cl2—Bi1—Cl1^i^	91.88 (13)	C1—C2—H2C	122.8
Cl1—Bi1—Cl1^i^	170.9 (2)	C2—C3—C4	123.0 (10)
Cl2—Bi1—Cl3	92.80 (16)	C2—C3—H3	118.5
Cl1—Bi1—Cl3	94.06 (17)	C4—C3—H3	118.5
Cl1^i^—Bi1—Cl3	94.06 (17)	C5—C4—C3	122.1 (10)
Cl2—Bi1—Cl4^ii^	87.32 (14)	C5—C4—H4	118.9
Cl1—Bi1—Cl4^ii^	85.93 (17)	C3—C4—H4	118.9
Cl1^i^—Bi1—Cl4^ii^	85.93 (17)	C4—C5—C6	116.5 (12)
Cl3—Bi1—Cl4^ii^	179.88 (13)	C4—C5—H5	121.8
Cl2—Bi1—Cl4	176.93 (13)	C6—C5—H5	121.8
Cl1—Bi1—Cl4	87.90 (13)	N3—C6—C1	104.7 (10)
Cl1^i^—Bi1—Cl4	87.90 (13)	N3—C6—C5	134.1 (13)
Cl3—Bi1—Cl4	90.27 (13)	C1—C6—C5	121.1 (13)
Cl4^ii^—Bi1—Cl4	89.61 (3)	N2—N3—C6	112.1 (10)
Bi1^iii^—Cl4—Bi1	157.68 (19)	N2—N3—H3A	123.9
H11—O1W—H22	106 (3)	C6—N3—H3A	123.9
N1—C1—C6	104.8 (10)	N2—N1—C1	112.0 (10)
N1—C1—C2	132.5 (10)	N2—N1—H1	124.0
C6—C1—C2	122.7 (10)	C1—N1—H1	124.0
C3—C2—C1	114.5 (10)	N3—N2—N1	106.4 (9)

Symmetry codes: (i) −*x*+1, *y*, *z*; (ii) −*x*+1, −*y*+1, *z*−1/2; (iii) −*x*+1, −*y*+1, *z*+1/2.

### Hydrogen-bond geometry (Å, º)

**Table:**

*D*—H···*A*	*D*—H	H···*A*	*D*···*A*	*D*—H···*A*
N1—H1···O1*W*	0.86	2.08	2.891 (17)	157
N3—H3*A*···O1*W*^iv^	0.86	2.00 (2)	2.767 (18)	148
O1*W*—H11···Cl4^iii^	0.89 (11)	2.47 (11)	3.306 (13)	157 (9)
O1*W*—H22···Cl3	0.90 (12)	2.38 (12)	3.268 (14)	169 (11)
C5—H5···Cl1^v^	0.93	2.73	3.603 (14)	157

Symmetry codes: (iii) −*x*+1, −*y*+1, *z*+1/2; (iv) −*x*+3/2, −*y*+1/2, *z*+1/2; (v) *x*+1/2, *y*−1/2, *z*+1.
